# Higher sensitivity monitoring of reactions to COVID-19 vaccination using smartwatches

**DOI:** 10.1038/s41746-022-00683-w

**Published:** 2022-09-09

**Authors:** Grace Guan, Merav Mofaz, Gary Qian, Tal Patalon, Erez Shmueli, Dan Yamin, Margaret L. Brandeau

**Affiliations:** 1 Department of Management Science and Engineering, Stanford University, Stanford, CA USA; 2 Department of Industrial Engineering, Tel Aviv University, Tel Aviv, Israel; 3 Kahn Sagol Maccabi (KSM) Research & Innovation Center, Maccabi Healthcare Services, Tel Aviv, Israel

**Keywords:** Public health, Disease prevention, Infectious diseases

## Abstract

More than 12 billion COVID-19 vaccination shots have been administered as of August 2022, but information from active surveillance about vaccine safety is limited. Surveillance is generally based on self-reporting, making the monitoring process subjective. We study participants in Israel who received their second or third Pfizer BioNTech COVID-19 vaccination. All participants wore a Garmin Vivosmart 4 smartwatch and completed a daily questionnaire via smartphone. We compare post-vaccination smartwatch heart rate data and a Garmin-computed stress measure based on heart rate variability with data from the patient questionnaires. Using a mixed effects panel regression to remove participant-level fixed and random effects, we identify considerable changes in smartwatch measures in the 72 h post-vaccination even among participants who reported no side effects in the questionnaire. Wearable devices were more sensitive than questionnaires in determining when participants returned to baseline levels. We conclude that wearable devices can detect physiological responses following vaccination that may not be captured by patient self-reporting. More broadly, the ubiquity of smartwatches provides an opportunity to gather improved data on patient health, including active surveillance of vaccine safety.

## Introduction

Vaccines are among the most effective tools available for preventing disease and death^1^ (https://www.cdc.gov/vaccines/adults/vpd.html). Currently, the world’s largest vaccination campaign ever is underway, with more than 12 billion COVID-19 vaccination shots administered by August 2022 (https://www.bloomberg.com/graphics/covid-vaccine-tracker-global-distribution/). The accelerated speed at which COVID-19 vaccines were developed was unprecedented and has led to concerns about vaccine efficacy, safety, and side effects^2^.

Many efforts are underway to monitor vaccine side effects, though the currently used self-reporting methods may lead to biased data. For example, data from health care providers and public agencies on COVID-19 vaccine side effects is gathered by the World Health Organization through its Adverse Event Following Immunization database (http://investigation.gvsi-aefi-tools.org/), by the European Union through its EudraVigilance database (https://www.adrreports.eu/en/index.html), and in the U.S. through its Vaccine Adverse Event Reporting System (https://www.fda.gov/vaccines-blood-biologics/vaccine-adverse-events/vaers-overview). Current guidelines for evaluating the safety of vaccines are primarily based on self-reports in electronic diaries, which by nature of being a questionnaire can introduce potential bias due to inaccuracies in self-reporting, non-continuous data, and lack of sensitivity^3–5^. Some surveillance information is captured directly from vaccinated individuals through smartphone questionnaires. For example, the U.S. Centers for Disease Control and Prevention (CDC) has developed a smartphone app that allows vaccinated individuals to report any side effects they experience (https://www.cdc.gov/coronavirus/2019-ncov/vaccines/safety/vsafe.html).

An additional, more objective source of information on COVID-19 vaccine side effects is data from wearable devices, such as smartwatches. Worn by approximately 20% of the U.S. population (https://www.prweb.com/releases/u_s_smartwatch_sales_see_strong_gains_according_to_new_npd_report/prweb16094466.htm; https://www.pewresearch.org/fact-tank/2020/01/09/about-one-in-five-americans-use-a-smart-watch-or-fitness-tracker/), wearable devices are a promising technology for healthcare applications as they can continuously monitor physiological measures such as an individual’s heart rate, oxygen saturation, and physical activity^6^. Recent research has shown that smartwatches may identify physiological changes undetected by the individual. For example, wearable devices have been recently shown to be useful in detecting early signs of COVID-19 symptoms^7–11^ as well as long-term effects of COVID-19 infections^12^.

In this study, we sought to determine whether smartwatches can be more sensitive than self-reported questionnaires in detecting COVID-19 vaccine side effects in a large sample size. Specifically, we examined post-vaccination Garmin Vivosmart 4 smartwatch data from two cohorts of individuals in Israel who received the second and third (booster) COVID-19 vaccine doses, respectively. We assessed potential side effects for up to 336 h (14 days) post-vaccination, focusing on the first 72 h post-vaccination. We compared these findings with self-reported questionnaires filled out by these individuals.

## Results

### Cohort characteristics

Table 1 provides a description of the cohorts. The youngest and oldest participants were 18 and 88 years old, respectively. The average participant age was 51.8 years and 50.0 years for the cohorts receiving the second and third vaccination, respectively, with the two cohorts comprising 57.9% women and 56.6% women, respectively. In the two cohorts, 24.8% and 38.8% of participants, respectively, reported having a comorbid condition.

**Table 1:** Description of cohort participants and self-reported reaction severity after second and third vaccinations.

	Second vaccination, no reaction	Second vaccination, mild reaction	Second vaccination, severe reaction	Third vaccination, no reaction	Third vaccination, mild reaction	Third vaccination, severe reaction
Total (%)	181 (54.0%)	102 (30.4%)	52 (15.6%)	655 (55.6%)	404 (34.2%)	120 (10.2%)
*Sex*						
Female (%)	96 (53.0%)	61 (59.8%)	37 (71.2%)	355 (54.2%)	235 (58.2%)	77 (64.2%)
Male (%)	85 (47.0%)	41 (40.2%)	15 (28.8%)	300 (45.8%)	169 (41.8%)	43 (35.8%)
*Age (years)*						
Mean	57.46	45.26	44.71	52.38	47.55	44.84
Std	14.21	14.28	15.92	15.07	15.53	13.93
Range	22–88	22–79	23–80	20–86	18–88	20–74
Median	60.0	42.5	42.5	56.0	51.0	44.5
*Age range*						
18–55 (%)	69 (38.1%)	79 (77.5%)	37 (71.2%)	326 (49.8%)	256 (63.4%)	87 (72.5%)
>55 (%)	112 (61.9%)	23 (22.5%)	15 (28.8%)	329 (50.2%)	148 (36.6%)	33 (27.5%)
*Underlying medical condition*						
Yes (%)	61 (34.3%)	14 (14.4%)	8 (15.7%)	278 (42.6%)	141 (35.1%)	38 (31.7%)
No (%)	117 (65.7%)	83 (85.6%)	43 (84.3%)	374 (57.4%)	261 (64.9%)	82 (68.3%)
Not reported	3	5	1	3	2	0

### Questionnaire analysis

In the questionnaire, 46.0% and 44.4% of participants reported side effects (i.e., mild or severe symptoms) after the second and third vaccinations, respectively (Table 1). Females reported greater rates of side effects than males: 50.5% vs. 39.7% after the second vaccination and 46.8% vs. 41.4% after the third vaccination. Participants younger than 55 were more likely to report experiencing side effects than participants older than 55: 62.7% vs. 25.3% after the second vaccination, and 51.3% vs. 35.5% after the third vaccination.

**Fig. 1:**
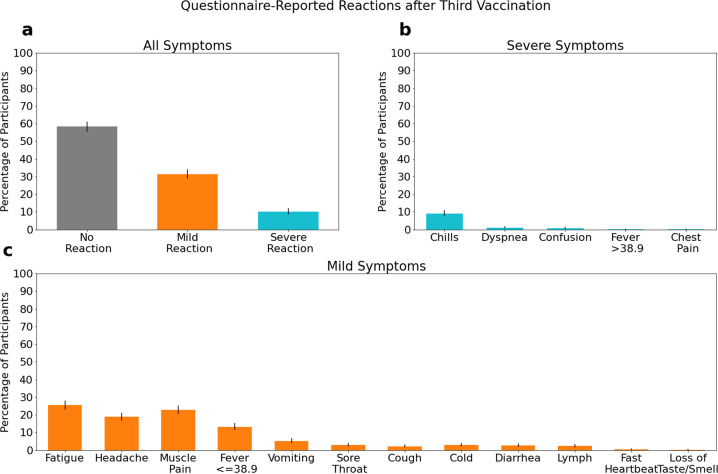
Summary of symptoms as reported in the questionnaires after the third vaccination. Percentage of all participants classified into each severity tier based on their most severe reported symptom in the 72 h following vaccination (**a**), percentage of all participants reporting each of the severe symptoms (**b**), and percentage of patients reporting each of the mild symptoms (**c**). Error bars represent 95% confidence intervals.

Similar to prior studies of the BNT162b2mRNA vaccine^4^ (https://www.medrxiv.org/content/10.1101/2021.05.06.21256587v1), the mild symptoms of fatigue, headache, and muscle pain were the most reported reactions (Fig. 1, Supplementary Fig. 2). Fewer than half of participants reported any side effects after the second and third vaccinations, and fewer than 16% of participants reported experiencing severe symptoms after either vaccination (Table 1). Although the self-reported questionnaires were not validated clinically (by medical examination of participants), the symptom severity in the questionnaire largely aligned with effects on heart rate in the 72 h post vaccination. Moreover, the reaction types, frequency, and duration we observed for the second dose were similar to those observed in the 340 BNT162b2 mRNA vaccine clinical trial^4^.

### Wearables analysis

**Fig. 2:**
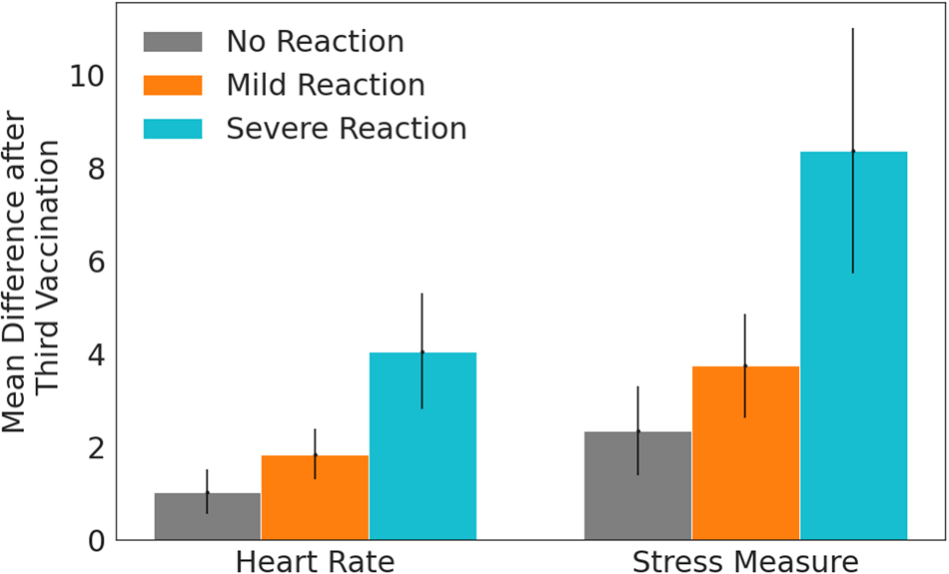
Change in heart rate and stress measure as a function of reaction severity. Mean difference in heart rate (in beats per minute) and stress measure (in points) between the post-vaccination and baseline periods in Garmin smartwatch data in the 72 h after the third vaccination, stratified by self-reported reaction severity. Error bars represent 95% confidence intervals.

We analyzed the difference in heart rate and stress measure averaged over the first 72 h post vaccination, finding that after the third vaccination, those who reported a severe reaction had significantly higher mean difference in heart rate and stress measure compared to baseline than those who reported mild (*p*-value < 1.77 × 10^−3^ for heart rate, <1.92 × 10^−3^ for stress measure) or no reactions (*p*-value < 2.16 × 10^−5^ for heart rate, <5.17 × 10^−5^ for stress measure) (all p-values reflect t-tests for the means of two independent samples with unequal variance) (Fig. 2). After the second vaccination, we see a similar trend in that the average heart rate was higher in participants with severe reactions compared to those with mild or no reactions; however, this difference was not significant (mild reaction *p*-value = 0.381 for heart rate, = 0.442 for stress measure; no reaction *p*-value = 0.077 for heart rate, = 0.070 for stress measure) (Supplementary Fig. 3). The magnitude of this difference as captured by the smartwatches accurately reflected the classified severity of reactions self-reported by participants (no reaction, mild, severe).

**Fig. 3:**
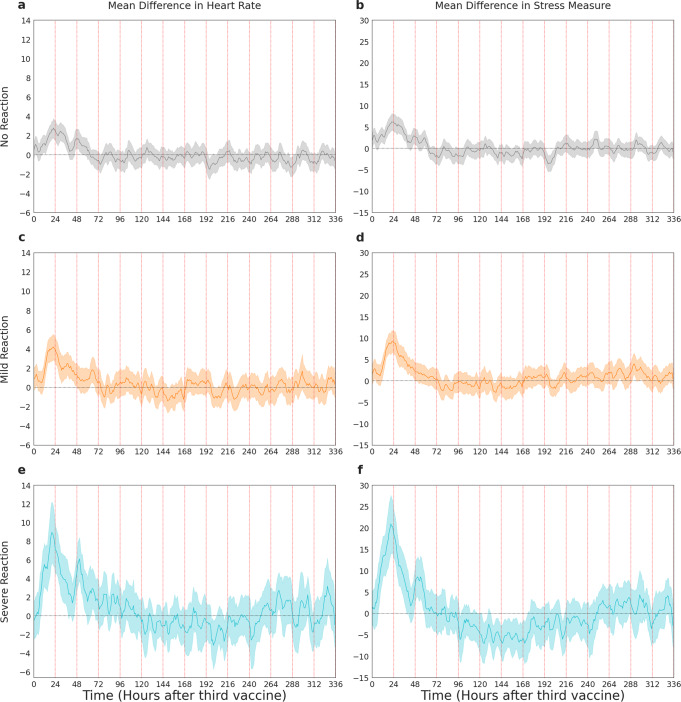
Change in heart rate and reaction severity for each hour after vaccination. Mean difference in heart rate (in beats per minute) and stress measure (in points) between the post-vaccination and baseline periods in Garmin smartwatch data after the third vaccination, by hour, for individuals who reported no reaction (**a** and **b**), mild reaction (**c** and **d**), and severe reaction (**e** and **f**) in the self-reported questionnaires. Shaded regions represent 95% confidence intervals.

For participants who reported no reaction after the second or third vaccinations, the smartwatches detected statistically significant physiological effects of vaccination as compared to the baseline period. Specifically, among participants who reported no reaction to the third vaccination, the wearables detected significant elevation in heart rate and stress measure (Fig. 3a, b). For these participants, their mean difference in heart rate compared to baseline peaked at 2.77 more beats per minute (95% CI [1.79, 3.75], *n* = 348) and their mean difference in stress measure peaked at 6.21 points higher (95% CI [4.31, 8.11], *n* = 336) after the third vaccination compared to their corresponding values during the baseline period. Participants returned to baseline by 72 h after the third vaccination. We found a similar result after the second vaccination among participants who reported no reaction (Supplementary Fig. 4a, b): compared to their corresponding values during the baseline period, mean difference in participants’ heart rates on average peaked at 3.50 more beats per minute (95% CI [1.50, 5.50], *n* = 63) higher, and mean difference in participants’ stress measures on average peaked at 6.77 points higher (95% CI [2.86, 10.68], *n* = 60). The scale of the difference in stress measure can be partially deduced by our stratification of reaction severity. For example, after the third vaccination, a mean difference of 2.345 (95% CI [1.391, 3.298], *n* = 434) was associated with no reaction, 3.746 (95% CI [2.623, 4.869], *n* = 267) was associated with a mild reaction, and 8.374 (95% CI [5.728, 11.019], *n* = 76) was associated with a severe reaction (Fig. 2).

Wearable devices were more sensitive than the questionnaires in allowing us to determine when participants returned to their baseline levels. For participants who reported severe reactions, their corresponding measures of heart rate and stress after the third vaccination respectively peaked at increases of 8.91 more beats per minute (95% CI [5.64, 12.18], *n* = 63) and 20.91 points higher (95% CI [14.19, 27.63], *n* = 59) compared to baseline (Fig. 3e, f). While the reported level of severe symptoms of participants who reported a severe reaction returned to baseline levels after 72 h post-vaccination (see analysis in Supplementary Methods and Supplementary Fig. 5), the smartwatches detected that this return to baseline took longer than reported. Among participants who reported a severe reaction to the third vaccination, their stress level as measured by the smartwatch increased after vaccination, then decreased to below-baseline levels, and only returned to baseline by 11 days after the third vaccination (Fig. 3f). Participants who reported mild reactions returned to baseline within 72 h after vaccination for both the second and third vaccine doses (Fig. 3c, d, Supplementary Fig. 4c, d).

### Panel regression

Table 2 presents the results of our mixed effects panel regressions after the third vaccination. For the regression analyzing heart rate, the coefficient for the indicator variable of being between 0 and 72 h after vaccination was significantly positive both for the regression over the entire cohort (coefficient = 0.7232, t-test *p*-value < 0.001) and for only asymptomatic participants (coefficient = 0.4922, t-test *p*-value < 0.001). Random effects accounted for 8.38% and 7.35% of the variability in those models, respectively. Similarly, for the regression analyzing stress measure after the third vaccination, the coefficient for the indicator variable of being between 0 and 72 h after vaccination was significantly positive both for the regression over the entire cohort (coefficient = 0.8997, t-test *p*-value < 0.001) and for only asymptomatic participants (coefficient = 0.6225, t-test *p*-value < 0.001). Random effects accounted for 3.54% and 4.15% of the variability in those models, respectively. As expected, the effect of vaccination was smaller for participants reporting no symptoms in the questionnaire compared to all participants: the coefficient for the regression for asymptomatic participants was lower than the coefficient for the regression over all participants for both heart rate and stress measure.

**Table 2:** Results of the mixed effects panel regression for all participants and asymptomatic participants only after the third vaccination. *P*-values for variable coefficients are from two-sided t-tests, and p-values for F-statistics are from F-tests.

Dependent Variable: Heart Rate (HR)^a^		
Fixed and Random Effects	All Participants (*n* = 1070) Coefficient, Std Err, *p*-value	Asymptomatic Participants Only (*n* = 523) Coefficient, Std Err, p-value
Between 0 and 72 h after vaccination	0.7232, 0.0427, <0.001	0.4922, 0.0607, <0.001
HR or Stress in Previous Hour	0.4872, 0.0012, <0.001	0.4802, 0.0018, <0.001
Age	−0.1651, 0.002, <0.001	−0.1638, 0.003, <0.001
No Underlying Medical Condition	−1.4746, 0.056, <0.001	−1.6324, 0.078, <0.001
Male Gender	−3.2402, 0.053, <0.001	−2.7722, 0.075, <0.001
	F-statistic = 79220 (*p*-value < 0.0001)	F-statistic = 37600 (*p*-value < 0.0001)
	R^2^ Between = 0.6718	R^2^ Between = 0.6635
	R^2^ Within = 0.3481	R^2^ Within = 0.3399
	R^2^ Overall = 0.4535	R^2^ Overall = 0.4449
	% of Variance due to Random Effects: 8.38%	% of Variance due to Random Effects: 7.35%
Dependent Variable: Stress Measure^a^		
Fixed and Random Effects	All Participants (*n* = 1063) Coefficient, Std Err, *p*-value	Asymptomatic Participants Only (*n* = 519) Coefficient, Std Err, *p*-value
Between 0 and 72 h after vaccination	0.8997, 0.0615, <0.001	0.6225, 0.0873, <0.001
HR or Stress in Previous Hour	0.7392, 0.0012, <0.001	0.7364, 0.0018, <0.001
Age	−0.1748, 0.003, <0.001	−0.1719, 0.004, <0.001
No Underlying Medical Condition	0.9068, 0.086, <0.001	1.7162, 0.12, <0.001
Male Gender	1.1384, 0.081, <0.001	1.1123, 0.116, <0.001
	F-statistic = 183200 (p-value < 0.0001)	F-statistic = 87710 (p-value < 0.0001)
	R^2^ Between = 0.8315	R^2^ Between = 0.8198
	R^2^ Within = 0.5595	R^2^ Within = 0.5550
	R^2^ Overall = 0.5865	R^2^ Overall = 0.5827
	% of Variance due to Random Effects: 3.54%	% of Variance due to Random Effects: 4.16%

^a^The number of participants for the heart rate and stress measure panel regression differ slightly due to issues extracting the data from Garmin.

We found similar results after the second vaccination despite the smaller sample size (Supplementary Table 1). The coefficient for the indicator variable of being between 0 and 72 h after vaccination in the regression analyzing heart rate was significantly positive both for the regression over the entire cohort (coefficient = 0.7331, t-test *p*-value < 0.001) and for only asymptomatic participants (coefficient = 0.4007, t-test *p*-value = 0.0051). Random effects accounted for 6.14% and 3.43% of the variability in those models, respectively. Similarly, with stress measure as the dependent variable after the second vaccination, the coefficient for the indicator variable of being between 0 and 72 h after vaccination was significantly positive both for the regression over the entire cohort (coefficient = 0.6895, t-test *p*-value < 0.001) and for only asymptomatic participants (coefficient = 0.5793, t-test *p*-value = 0.0046). Random effects accounted for 0.29% and 2.25% of the variability in those models, respectively. Similar to after the third vaccination, the effect of vaccination was smaller for participants reporting no symptoms in the questionnaire compared to all participants; this is also reflected in the higher, but still significant, p-values in the regressions considering only asymptomatic participants.

Overall, for both dependent variables of heart rate and stress measure, both after the second and third vaccinations, all coefficients for the indicator variable of being between 0 and 72 h after vaccination were significant, even after applying the Bonferroni correction for multiple comparisons (lowering the significance threshold to 0.008). Importantly, even for participants who reported no symptoms in the questionnaire following vaccination, we were able to identify a significant elevation in heart rate and stress between 0 and 72 h after vaccination. This effect was not explained by age, underlying medical condition, gender, or other participant-level random effects.

## Discussion

Our analysis of smartwatch data to monitor reactions to the second and third BNT162b2mRNA COVID-19 vaccinations has demonstrated that wearable device data allows for greater sensitivity than using self-reported questionnaires alone. We found that the elevation in heart rate and stress measure post-vaccination was positively correlated with the severity and duration of the reported symptoms. These measures are of interest because they provide in an objective manner continuous information on two major systems of the human body: the cardiovascular system and the nervous system. Moreover, these measures allowed greater sensitivity than using self-reported questionnaires alone—which is currently the primary mechanism for evaluating the safety of vaccines. Importantly, even among participants who did not report side effects after vaccination, the smartwatch detected a significant physiological response compared to the baseline period in the first 72 h after vaccination (t-test *p*-value < 0.005), even after removing participant-level fixed and random effects. While participants returned to their baseline levels on average 72 h after vaccination, analysis of smartwatch stress data reveals that participants who had a severe reaction to the third vaccination, as categorized by symptoms self-reported in the questionnaire, took longer to return to their baseline levels.

This work is part of a growing stream of literature using consumer-grade smartwatches to assess interventions. Our contribution is that we combine wearable device data with information about participants’ vaccine side effects from a questionnaire to demonstrate the greater sensitivity of wearable devices. Other studies analyzing COVID-19 vaccine side effects may not use consumer-grade wearable devices, for example using questionnaires or chest patch sensors^13,14^. Furthermore, studies using consumer-grade wearable devices to analyze the effects of vaccination or other interventions do not analyze measures as granularly as we did; for example, one study in South Korea used smartwatches to assess the effect of the first and second COVID-19 vaccination on resting heart rate in a cohort of 7,728 individuals^15^. Our work is distinct in that we assess both our entire cohort of participants and only those participants who reported no side effects in a questionnaire. We find substantial increases in both heart rate and stress measure, which can be analyzed as hourly measurements rather than the daily measurement of resting heart rate. Furthermore, we use a random effects model to untangle other participant-level effects from the fixed effects of age, gender, and whether the participant has an underlying medical condition.

Our analysis has several limitations. The cohorts we studied are slightly older than the Israeli population, so our analyses may not generalize to the entire Israeli or global population. However, the most reported reactions and the frequency and duration of side effects for both cohorts were consistent with other clinical trial observations on the BNT162b2mRNA vaccine^4^. We only considered the BNT162b2mRNA vaccine since this is the only vaccine available in Israel. Results may be similar for other COVID-19 vaccines due to their similar profiles^4,16^.

Additionally, the questionnaire data could be biased. Participants did not always fill out the questionnaire each day and it is unknown whether or how the missing data could bias the results: as an example, if severity of side effects influenced whether an individual filled out the questionnaire on a given day, this could skew our results. Not all participants who reported feeling feverish reported their temperature, which could bias results as individuals with a fever who could be classified as having severe symptoms may have been categorized as having mild symptoms. Similarly, patients were not able to report subjective severity of symptoms in the questionnaire: for example, muscle pain could be mild or severe. These shortcomings in self-reported data highlight the need for objective physiological measures such as those obtained from smartwatches.

Many factors not associated with vaccination (e.g., caffeine or alcohol consumption) could influence heart rate measures. We assumed that for each participant these behaviors would be the same throughout the pre- and post-vaccination periods, so analysis of the mean difference between the two time periods does account for these factors. Moreover, the Garmin smartwatches that were used to obtain physiological measures of participants are not medical-grade wearable devices, nor are smartwatches representative of all wearable devices. Our study provides insight into the effectiveness of using off-the-shelf smartwatches to supplement questionnaires for health monitoring. We note that previous studies have demonstrated the accuracy of smartwatches in measuring heart rates^17,18^. Rather than focusing on absolute measurements, we compared heart rate measurements to a baseline collected using the same device. Lastly, we have shown that COVID-19 vaccination increases the Garmin-computed stress measure in the 72 h after vaccination compared to baseline. It is not possible to unambiguously assign causality to the vaccination as we did not explicitly control for the effects of the observational trial setting (i.e., participating in a trial, wearing a smartwatch, potential concerns regarding the vaccine, etc.). Any effects of the observational trial setting should, in principle, have similar impacts on our analysis of each of the three vaccine doses. However, two previous studies found no deviations in most measurements from baseline levels in the subset of participants who received their first dose^13,19^. Furthermore, in the current study, stress and heart rate were close to baseline levels during the first 12 h post-vaccination, supporting our assumption that the increases in heart rate and stress measure were a physiological response to the vaccination rather than a response to the medical encounter. Specifically, in the first 12 h after the third vaccination, heart rate increased on average by 0.67 beats per minute in participants who reported no reaction, 1.09 beats per minute in participants who reported a mild reaction, and 1.86 beats per minute in participants who reported a severe reaction compared to baseline levels, and stress measure increased on average by 2.28 points in participants who reported no reaction, 2.30 points in participants who reported a mild reaction, and 5.83 points in participants who reported a severe reaction compared to baseline levels, much smaller than the peaks observed around 24 h post-vaccination.

While our work may further support the safety of the second and third COVID-19 BNT162b2 mRNA vaccine dose from both a subjective and an objective perspective^4,20,21^, the clinical safety of the vaccine cannot be determined from this work. However, our study provides a proof of concept for the potential of smartwatches in reshaping clinical trials. Future studies should be conducted to properly define clinical safety based on smartwatch measures.

Wearable devices allow for continuous assessment of physiological measures post-vaccination. Now, as vaccine booster shots are increasingly being recommended (https://www.cdc.gov/media/releases/2021/p1021-covid-booster.html) and the possibility has arisen that individuals may need to be vaccinated annually against COVID-19 (https://www.businessinsider.com/pfizer-ceo-albert-bourla-predicts-annual-covid-19-boosters-shots-2021-8), wearable devices can provide valuable data about side effects of the vaccination. Future work could apply the framework of our analysis to other diseases or health conditions for which continuous monitoring of a patient’s physiological state can augment patient self-reports – with or without a breakpoint such as vaccination. Future work could also analyze self-reports in conjunction with wearable devices, for example, to assess whether certain questionnaire responses (e.g., pain) are more associated with wearable device measures (e.g., heart rate) than others.

Wearable devices, particularly smartwatches, are widely used, and the market for such devices is growing rapidly (https://www.grandviewresearch.com/industry-analysis/wearable-medical-devices-market). Because of their potential to provide continuous measurements of critical biomarkers, wearable devices are increasingly used in a variety of applications in areas such as real-time health monitoring, disease diagnosis, prediction, and prevention, and personalized medicine^6,22^. More broadly, the considerably higher sensitivity of wearable sensors can revolutionize clinical trials by enabling earlier identification of abnormal reactions, potentially allowing for fewer subjects.

## Methods

### Cohort

We studied cohorts from a prospective observational trial of 355 and 1,179 individuals who received their second and third vaccinations, respectively, in Israel with the BNT162b2 mRNA (Pfizer BioNTech) COVID-19 vaccine between January 10, 2021 and September 15, 2021. The cohorts were mostly disjoint, with 116 individuals in both cohorts. Upon enrollment in the study, we collected information on participants’ gender, age, and underlying medical conditions, which consisted of hypertension, diabetes, heart disease, chronic lung disease, immune suppression, cancer, renal failure, body mass index (BMI) > 30 (BMI is defined as weight in kilograms divided by the square of height in meters). We focused on analyzing side effects after the second and third vaccinations.

### Study design

Participants filled out a daily questionnaire on the PerMed mobile application^23^. The questionnaire collected self-reports from individuals on clinical symptoms from a list of reactions observed in the BNT162b2 mRNA Covid-19 vaccine clinical trial^4^, with an option to add other symptoms as free text (Supplementary Fig. 1). The self-reported questionnaires were formulated by the study team and clinicians. The questions were formulated based on potential signs and symptoms following infection with infectious diseases and respiratory infections; we examined ICD9 codes for influenza, and influenza-like illness, acute respiratory infections, RSV, group A streptococcus, and COVID-19. A pilot study was conducted between May 11, 2020 and October 17, 2020 with 192 participants to ensure that the questions are clear and that there is consistency between symptoms and smartwatch measures. Various results from the self-reported questionnaires appear elsewhere^13,19,23^.

Participants wore a Garmin Vivosmart 4 smartwatch beginning when they were recruited into the study and for the duration of the study. Data from the smartwatch was used to estimate the effects of the vaccine on the physiological measures of heart rate and heart rate variability-based stress (“stress measure”). Stress is a measure between 1 to 100 computed by Garmin and is categorized into four tiers: rest (1–25), low (26–50), medium (51–75), and high (76–100) (https://support.garmin.com/en-US/?faq=WT9BmhjacO4ZpxbCc0EKn9). Higher stress measure is associated with lower heart rate variability; this connection is supported by previous studies^24,25^. Heart rate data (beats per minute) was provided in 15-s increments. Stress measure data was given in 3-min increments.

We implemented several measures to minimize attrition and churn of participants and consequently improve the quality, continuity, and reliability of the collected data. First, each day, if by 7 pm, participants had not yet filled out the daily questionnaire, they received a reminder notification through the PerMed application. During peak periods of COVID-19 vaccination in Israel, we also increased the frequency of the reminders and adjusted their content. Second, we developed a dedicated dashboard that allowed the survey company to identify participants who continually neglected to complete the daily questionnaires or did not wear their smartwatch for a long period of time; these participants were contacted by the survey company (either by text messages or phone calls) and were encouraged to better adhere to the study protocol. Third, to strengthen participants’ engagement, a weekly personalized summary report was generated for each participant and was available inside the PerMed application. Similarly, a monthly newsletter with recent findings from the study and useful tips regarding the smartwatch’s capabilities was sent to the participants. At the end of the study, participants will receive all personal insights that were obtained and can keep the smartwatch as a gift.

Further information regarding the recruitment procedure, choice of smartwatch data analyzed, data collection architecture, and PerMed dashboard is provided in the Supplement.

### Statistical analysis

#### Preprocessing

Though most participants filled out the questionnaire at most once per day, if a participant submitted multiple questionnaire entries in a day, we considered only the participant’s last questionnaire entry on that day; we assumed that the last entry was most reflective of the participant’s entire day. Our rationale was that questionnaires could not be updated after being sent to the server; in case of a filling error, participants were instructed to submit a new questionnaire.

#### Baseline period

We defined a “baseline period” of 7 days prior to vaccination. We considered a participant’s “baseline” to be the last questionnaire they filled out during the baseline period and smartwatch data for the entire baseline period. If a symptom was reported after vaccine administration and was not reported during the baseline period, we assumed this was a vaccine side effect. If participants did not fill out the questionnaire during the baseline period, they were excluded from our analysis since we could not tell if their symptoms also existed prior to vaccination. We compared the baseline period to the “post-vaccination period,” which we defined as 7 and 14 days after vaccination, inclusive of vaccination day, for discrete and continuous metrics, respectively. Since the U.S. CDC states that vaccine side effects should disappear after a few days, we focused our analysis on the first 72 h post-vaccination (https://www.cdc.gov/coronavirus/2019-ncov/vaccines/expect/after.html).

#### Inclusion of participants

We included participants who (1) submitted at least one questionnaire during the baseline period, (2) submitted at least one questionnaire during the 72 h after vaccination, and (3) provided wearable device data during the same day-of-week and time-of-day during their post-vaccination and baseline periods.

We required at least one questionnaire during the baseline period, since if a participant did not fill out the questionnaire during the baseline period, we could not tell if their symptoms also existed prior to vaccination. We required at least one questionnaire during the 72 h after vaccination to understand the self-reported severity of the participant’s reaction to the vaccination. Lastly, we required wearable device data during the same day-of-week and time-of-day during their post-vaccination and baseline periods to compute the difference between those two time periods for our analysis.

If a participant only provided data for only one day (during either the baseline or post-vaccination period), we did not include that participant. If a participant provided data for the same hours-of-day and days-of-week during the baseline and post-vaccination periods, we included them in our analysis for the periods of time for which they provided data, even if only one day (the same day-of-week and hours-of-day) during each period was given.

#### Severity of reaction

We stratified participants by the severity of the reactions they reported in the questionnaire in the 72 h after each vaccination. For participants who reported feeling hot and recorded their temperature, we classified the temperature as above 38.9 °C (fever) or below 38.9 °C (feeling hot); if the participant did not record their temperature, we classified the temperature as below 38.9 °C.

Based on the CDC (https://www.cdc.gov/coronavirus/2019-ncov/vaccines/different-vaccines/Pfizer-BioNTech.html) and the Pfizer clinical trial^4^, we categorized symptoms as follows:

- Mild symptoms: abdominal pain, feeling hot, back or neck pain, feeling cold, muscle pain, weakness, headache, dizziness, vomiting, sore throat, diarrhea, cough, leg pain, ear pain, loss of taste and smell, swelling of the lymph nodes, fast heartbeat, and hypertension;
- Severe symptoms: chest pain, dyspnea (shortness of breath), fever, confusion, and chills.

Participants were either classified as having “No Reaction,” a “Mild Reaction,” or a “Severe Reaction,” based on their most severe symptom reported in the 72 h after each vaccination. Thus, if a participant reported one severe symptom for one day and mild symptoms for all three days after vaccination, the participant was classified as having a severe reaction. Participants could be categorized into different severity groups after each vaccination dose.

#### Data analysis

From the questionnaire data, we computed the proportion and corresponding 95% confidence interval (CI) of participants who reported experiencing each side effect in the post-vaccination period. The 95% CI for each side effect was calculated using a beta distribution Beta(*α, β*), where *α* = number of participants reporting the symptom, and *β* = number of participants not reporting the symptom. We chose the beta distribution to represent this proportion since the distribution is defined over [0, 1].

For the continuous Garmin smartwatch measurements for heart rate and stress measure, we performed day-of-week and hour-of-day comparisons on the individual level, between the 14 days inclusive of vaccination day and the participant’s baseline period (7 days prior to vaccination). For example, we would compare a participant’s Tuesday average 10 pm heart rate and stress measure with their previous Tuesday average 10 pm heart rate and stress measure. Because Garmin smartwatches measure heart rate and stress data in different increments, we first computed the average value for each participant for each hour post-vaccination. Some discontinuities in the data were present (e.g., when a participant took off the smartwatch to charge it). All discontinuities with lengths fewer than 5 h were linearly interpolated. Participants who had more than 5 continuous hours of missing data were excluded from analysis during the time periods when they were missing data. We smoothed the data by calculating the five-hour moving average. We then calculated the mean difference over participants between the post-vaccination period and the corresponding baseline period. Finally, we also computed the 95% confidence intervals for these metrics.

To compare mean differences in heart rate and stress measure between groups of participants, we performed a two-sided Welch’s t-test, which does not assume equal population variance.

#### Panel regression

To remove participant-level effects from the effect of vaccination when analyzing heart rate and stress measure, we used a mixed effects panel regression with participants as random effects. Each observation in the panel regression reflected a participant’s measurements for a given hour for 168 h before and 168 h after vaccination (336 maximum time measurements per participant). We used the linearly interpolated but not smoothed data in this analysis. Dependent variables were hourly average heart rate and stress measure. Independent variables included age, gender, whether or not the participant had an underlying medical condition, one-hour lagged heart rate or stress measure, and an indicator variable for whether the data point was taken between 0 and 72 h after vaccination. Age, gender, and whether or not the participant had an underlying medical condition were time-invariant fixed effects. In addition to these fixed effects, we included participants as random effects (see Supplement for details).

To investigate the relationship between vaccination and the dependent variables, we evaluated the significance of the coefficient for the indicator variable for whether the data point was taken between 0 and 72 h after vaccination. Our null hypothesis was that this coefficient was equal to 0; the alternate hypothesis was that this coefficient was greater than 0. We used a one-sided test since we wanted to determine whether there was an increase in heart rate or stress measure following vaccination. To correct our regression coefficients’ estimates for multiple comparisons, we applied the Bonferroni correction. Since we estimated six coefficients, our p-value to reject the null hypothesis was 0.008.

We performed these mixed effects panel regressions separately for the second and third vaccinations. After each vaccination, we considered both all participants and only the participants who reported no symptoms in the questionnaire following vaccination. We compared several models, choosing the model with the highest R^2^, and confirming that all models had robust F-values. Lastly, we performed sensitivity analysis by changing the post-vaccination indicator variable to reflect whether the data point was taken between 24 and 72 h after vaccination to determine whether the increased heart rate and stress were effects of vaccination and not of the medical procedure of vaccination (see Supplement for details).

### Reporting summary

Further information on research design is available in the Nature Research Reporting Summary linked to this article.

## Supplementary information

Supplementary Material

Reporting Summary

## Data Availability

The aggregated datasets analyzed for the study are publicly available at https://github.com/permedtau/covid-vaccines-smartwatch-sensitivity-paper.

## Code availability

The code written for the study is publicly available at https://github.com/permedtau/covid-vaccines-smartwatch-sensitivity-paper.
