# Supplementary material for: Higher sensitivity monitoring of reactions to COVID-19 vaccination using smartwatches
Source: NPJ Digit Med. 2022 Sep 9;5:140. doi: 10.1038/s41746-022-00683-w (PMC9461410; doi:10.1038/s41746-022-00683-w)
Supplement: Supplementary file 1 — Supplementary Material [file 41746_2022_683_MOESM1_ESM.pdf]

SUPPLEMENTARY INFORMATION – HIGHER SENSITIVITY MONITORING OF REACTIONS TO COVID-19 VACCINATION USING SMARTWATCHES

GUAN ET AL.

ITEM	DESCRIPTION
**Supplementary Methods**	Data Collection, Supplemental Analysis, and Study Protocol
**Supplementary Figure 1**	Screenshot from mobile questionnaire showing the selection of symptoms.
**Supplementary Figure 2**	Summary of symptoms reported in the self-reported questionnaires after the second vaccination.
**Supplementary Figure 3**	Mean difference in heart rate (in beats per minute) and stress measure (in points) between the post-vaccination and baseline periods in Garmin smartwatch data in the 72 hours after the second vaccination, stratified by self-reported reaction severity.
**Supplementary Figure 4**	Mean difference in heart rate (in beats per minute) and stress measure (in points) between the post-vaccination and baseline periods in Garmin smartwatch data after the second vaccination, by hour, for individuals who reported no reaction, mild reaction, and severe reaction in the self-reported questionnaires.
**Supplementary Figure 5**	Percentage of participants who experienced a severe reaction after the third vaccination who also reported at least one severe symptom in the days post-vaccination.
**Supplementary Figure 6**	Compliance with study protocol for 7 days prior to and 13 days after the third vaccination (where day 0 reflects the day of vaccination), reflecting the dates used in the panel regression.
**Supplementary Figure 7**	The high-level architecture of the PerMed's data collection platform.
**Supplementary Figure 8**	Average autocorrelation (top) and partial autocorrelation (bottom) between hourly average measurements of heart rate (left) and stress measure (right) after the third vaccination.
**Supplementary Table 1**	Results of the mixed effects panel regression for all participants and asymptomatic participants only after the second vaccination.
**Supplementary Table 2**	Percentage of participants complying with each step of the enrollment procedure as of September 15, 2021.
**Supplementary Table 3**	Results of the mixed effects panel regression for asymptomatic participants after the third vaccination where the indicator variable has been changed to exclude the day of vaccination.
**Supplementary Table 4**	Self-reported reaction severity after the second and third vaccinations for the 95 individuals who were in both cohorts and provided self-reported data.
**Supplementary References**	References

Supplementary Methods

Data Collection

Recruitment Procedure

We publicized information about the study on various social networks, such as Facebook, Instagram, Twitter, and other platforms in Hebrew. In the study announcement, interested individuals were referred to a dedicated site where we described the study in more detail. Those interested filled in their contact information (telephone number and email). Study researchers contacted interested individuals by telephone or by Zoom. The study team assessed whether interested individuals met inclusion criteria and described the study in detail to the participants. If the participant was interested and met the criteria (see Study Protocol in the Supplement), research coordinators set up a meeting with the participant and received both consent from the potential participant using a written or electronic consent form and approval to our privacy policy. Once the consent form was signed, the participant received the smartwatch and downloaded the PerMed mobile application.

As of September 15, 2021, the study included 2,129 active participants (**Supplementary Table 2**). We report compliance for the 7 days pre- and 13 days post- third vaccination, which includes the time frame that we used to run the panel regression in the main text (7 days pre- and post- third vaccination). Questionnaire compliance was measured by the percentage of participants completing the questionnaire each day, where the denominator is n=1,179 reported in **Table 1**. Since participants were required to fill out the questionnaire every 3 days, we note that compliance in each 3-day window from the 6 days before through the 6 days after vaccine was much higher, at 89%. Smartwatch compliance was measured by the average percentage of each day participants wear their smartwatches, as reflected by how many hours out of 24 we received raw Garmin data in each day. We excluded 51 participants who did not wear their smartwatches over the entire 14-day period analyzed in the panel regression. Daily questionnaire and smartwatch compliance are given in **Supplementary Figure 6** and are both relatively high given the requests of the study.

Choice of Smartwatch Data Analyzed

We chose to present heart rate and heart rate variability-based stress as these measures were previously shown to be indicative in describing hemodynamic and physiologic changes following COVID-19 infection or vaccination[1-3]. The heart rate variability is affected by the vagus nerve associated with the nervous system[4,5]. Together, these simple two measures are available in most

smartwatches. Heart rate and stress measure thus provide continuous information on the two major systems of the human body: the cardiovascular system and the nervous system.

The smartwatch does not track the GPS location of the participants. Raw accelerometer data and GPS locations are generally considered sensitive information. In accordance with the data minimization principle (a detailed Data Protection Impact Assessments (DPIA) and Data Management Plan of the study is available from the corresponding author upon request), we do not access to the raw accelerometer data. Instead, we have access to other measures that are based on the accelerometer, including step counts, sleep duration, and sleep level classification, including light, deep, REM, and awake periods. These measures all correlate with heart rate and stress measure levels.

Data Collection Architecture

The data collection platform contains several components that interact with each other (see **Supplementary Figure 7**). The smartwatches send the data to the Garmin Connect app on the smartphone, which then sends this data to Garmin's server. We collect the data from Garmin through its API (application programming interface). The components are as follows:

- **The PerMed application** – This application is installed on each participant's phone to collect sensors data and the self-reported daily questionnaires. It also handles the smartwatch pairing.
- **The smartwatch application** – This application (currently either Fitbit or Garmin) receives information from the smartwatch via Bluetooth and transmits it to the company's server. In addition, it provides a convenient interface for displaying the participant's smartwatch information.
- **The webserver** – The webserver handles the database connectivity using REST API pages. It enables the server to authenticate users as they launch the application and write records to the database. This server also hosts the dashboard pages, which assist in monitoring the quality of the information and controlling the experiment.
- **The database** – A MySQL server stores the sensors' raw data, the answers to the daily questionnaires, participants' personal information, and dashboard statistics.
- **Batch processes running on the server** – These processes perform three different tasks: (1) sending app notifications (daily reminder to fill the questionnaire), (2) aggregating raw data for dashboard statistics, (3) fetching data from the smartwatch server to be stored in the database server.

The PerMed Dashboard

Participants were recruited by a qualified external recruitment team headed by Tel Aviv University personnel. The team received limited information essential to the control the experiment. Thus, we developed a dedicated dashboard for monitoring the quality of the information and controlling the experiment. This dashboard aimed to identify data collection issues such as participants who did not fill the daily questionnaires or participants who did not charge the battery of their smartwatches. The dashboard also helped us identify problems that were not related to participants' cooperation, such as bugs in the mobile app. This identification allowed us to respond faster and provide timely solutions.

Supplemental Analysis

Panel Regression

We used a mixed effects panel regression to assess the effect of vaccination on heart rate and stress measure while removing participant-related effects. We ran the following regressions over all participants for whom we had data between 168 hours prior to and 168 hours post vaccination:

$$HR_{i,t} = \rho HR_{i,t-1} + \beta^T X_i + \gamma I(0 \leq t \leq 72) + \alpha_i + \epsilon_{i,t}$$

$$SM_{i,t} = \rho SM_{i,t-1} + \beta^T X_i + \gamma I(0 \leq t \leq 72) + \alpha_i + \epsilon_{i,t}$$

The index *i* refers to participant and the index *t* refers to time (in hours, where *t=0* reflects the time of vaccination). $HR_{i,t}$ and $SM_{i,t}$ reflect the dependent variable of hourly average heart rate and stress measure, respectively, which depend on both the participant and time. X_i refers to time-invariant independent fixed effects depending only on each participant: age (continuous), gender (binary), and whether or not the participant has an underlying medical condition (binary). ρ, β, and γ are coefficients. α_i represents the unobserved time-invariant individual random effect. $\varepsilon_{i,t}$ is the error term (residual). $I(0 \leq t \leq 72)$ reflects an indicator variable of whether we are between 0 and 72 hours post-vaccination. Thus, we are interested in the significance of $\gamma>0$.

In our panel regression, we included an independent variable one-hour lagged of heart rate and stress measure since we observed strong autocorrelation (Pearson's $r=0.72$, $r=0.74$) between each subsequent hourly measurement of heart rate and stress measure (**Supplementary Figure 8, top**). While there was also strong autocorrelation every 24 hours, partial autocorrelation revealed that this effect was primarily due to the lower order autocorrelations (**Supplementary Figure 8, bottom)**. Thus, we chose not to include 24-hour lagged heart rate and stress measure in our regression. We did not balance the regression to weight all participants equally because we wanted to assign more weight to participants who provided more data.

We chose not to use a diff-in-diff model, because we are not observing any control units after the time of vaccination. Similarly, we chose not to use an ARIMA model since we have multiple observations over a short period of time rather than one observation over a longer period.

Untangling the effect of the procedure of vaccination

In sensitivity analysis, we changed the indicator variable reflecting the time after vaccination to remove the day of vaccination, that is, our regressions took the form of

$$HR_{i,t} = \rho HR_{i,t-1} + \beta^T X_i + \gamma I(24 \leq t \leq 72) + \alpha_i + \epsilon_{i,t}$$

$$SM_{i,t} = \rho SM_{i,t-1} + \beta^T X_i + \gamma I(24 \leq t \leq 72) + \alpha_i + \epsilon_{i,t}$$

We ran this regression after the third vaccination. Results of this regression are given in **Supplementary Table 3**. The coefficients γ for the indicator variable $I(24 \leq t \leq 72)$ are still significant in both regressions.

Severe Reaction Participants' Return to Baseline after Third Vaccination

Individuals who were classified as having a severe reaction to the third vaccination still reported severe symptoms in days 3-13 after the third vaccination (**Supplementary Figure 5**). We assessed whether these severe symptoms were reported at a higher rate compared to a normal day in their life, where we defined a normal day as a day outside of the baseline and post-vaccination periods (i.e., not within 7 days before or 14 days after any of the three vaccinations). We found that the proportion of participants who experienced severe reactions who still reported severe symptoms in days 3-13 after the third vaccination was no greater than normal-day levels.

The percentage of normal days during which individuals who were classified as having a severe reaction to the third vaccination reported severe symptoms was 2.22% (95% CI [1.63%, 2.85%], n=2270). For days 3-14 after vaccination, the 95% confidence interval of the percentage of participants on each of days 3-13 who experienced a severe reaction after the third vaccination who also reported at least one severe symptom overlaps this normal-day level 95% confidence interval.

Overlapping cohort between the vaccinations

For the 95 individuals who were in both the second and third dose cohorts and provided questionnaire responses, we compared the severity of their self-reported reactions to the second and third vaccinations (**Supplementary Table 4**). Of these individuals, 64.2% (61 of 95) had the same severity of reaction to both vaccinations. The change in reaction severity was equal between the two vaccinations: 17.9% (17 of 95) had a more severe reaction to the third vaccination compared to the second, and 17.9% (17 of 95) had a more severe reaction to the second vaccination compared to the

third. Only 4.2% (4 of 95) participants had a severe reaction to either the second or third vaccination and no reaction to the other.

Study Protocol

Study Design

In this study we will analyze data that was already collected and will be collected as part of the PerMed study[6]. Participants in the PerMed study are recruited for a period of two years, during which they are equipped with a Garmin Vivosmart 4 smartwatches and are asked to wear them as much as they could. In addition, participants install two applications on their mobile phones: an application that passively collects data from the smartwatch and a dedicated mobile application which allows participants to fill a daily questionnaire and to report their vaccine date and specific hour.

Participants

The inclusion criteria for the PerMed study includes those aged > 18 years who carry a smartphone. Individuals who are not eligible to give and sign a consent form of their own free will are excluded, as are individuals who know with certainty that they will be outside of Israel for more than three months during a continuous period at any point during the two years following enrollment in the study. In this study, we will analyze the data of participants who reported receiving at least one dose of the BNT162b2 mRNA COVID-19 vaccine after joining the PerMed study. To recruit participants and ensure they complete all the study's requirements, we will hire a professional survey company. Potential participants will be recruited through advertisements in social media, online banners, and word-of-mouth. The survey company is responsible for guaranteeing the participants meet the study's requirements, in particular, that the questionnaires are filled daily, ensuring the smartwatches are charged constantly and worn properly, and assisting participants resolve technical problems.

Study procedures

Before participation in the study, all participants will be advised orally and in writing about the nature of the experiments and give written, informed consent. At this time, participants will be asked to complete an enrollment questionnaire that includes demographic information and health status. In addition, participants will be asked to install two applications on their mobile phones: an application that passively collects data from the smartwatch and the PerMed application, which allows participants to fill in the daily questionnaires. Participants will be given instructions regarding

the self-reported symptoms questionnaires and how to operate the smartwatch, which they will wear as much as they can.

Enrollment questionnaire

All participants will fill a one-time enrollment questionnaire that includes demographic questions and questions about the participant's health condition in general. Specifically, the questionnaire will include the following: age, gender, height, weight and underlying medical conditions (Listed in **Table 1**, main text). Other questions such as name, address, phone and email will be recorded and used by the survey company to contact the participants. The answers will be filled-in directly by the survey company to the study's secured dashboard.

Monitoring device

Participants will be equipped with Garmin Vivosmart 4 smart fitness trackers. Among other features, the smartwatch provides all-day heart rate and heart rate variability and during-night blood oxygen saturation level tracking capabilities (www.garmin.com).

The optical wrist heart rate (HR) monitor of the smartwatch is designed to continuously monitor a user's heart rate. The frequency at which heart rate is measured varies and may depend on the level of activity of the user: when the user starts an activity, the optical HR monitor's measurement frequency increases.

Since heart rate variability (HRV) is not easily accessible through Garmin's application programming interface (API), we use Garmin's stress level instead, which is calculated based on HRV. Specifically, the device uses heart rate data to determine the interval between each heartbeat. The variable length of time between each heartbeat is regulated by the body's autonomic nervous system. Less variability between beats correlates with higher stress levels, whereas an increase in variability indicates less stress (https://support.garmin.com/en-US/?faq=WT9BmhjacO4ZpxbCc0EKn9). A similar relationship between HRV and stress was also seen in other studies[7,8].

Examining the data collected in our study, we identified an HR sample roughly every 15 seconds, and an HRV sample every 180 seconds.

While the Garmin smartwatch provides state-of-the-art wrist monitoring, it is not a medical-grade device, and some readings may be inaccurate under certain circumstances, depending on factors such as the fit of the device and the type and intensity of the activity undertaken by a participant[9,10] (https://www.garmin.com/en-US/legal/atdisclaimer/).

Vaccination questionnaire

The vaccination questionnaire we will use includes the following question:

- COVID-19 vaccination – date, time and dose number.

Daily questionnaires

All participants will complete the daily self-reported questionnaire in a dedicated application (the PerMed mobile application). The daily questionnaire we will use includes the following question:

> Have you experienced one or more of the following symptoms in the last 24 hours?• My general feeling is good, and I have no symptoms• Heat measured above 37.5• Cough• Sore throat• Runny nose• Headache• Shortness of breath• Muscle aches• Weakness / fatigue• Diarrhea• Nausea / vomiting• Chills• Confusion• Loss of sense of taste / smell• Another symptom.

Data Storage

Data collected from the mobile phone application and from the smartwatches will be stored on a secure server within Tel Aviv University facilities. The server runs a CentOS operating system and is located in Software Engineering Building at Tel Aviv University. This server is protected behind the university's firewall and is not connected to external networks. In addition, a secure connection through an SSL protocol and a trusted certificate will be obtained for the transfer of information from the mobile phone application into the secured server.

Access will be restricted to investigators in the study. The information from the mobile application will be stored in a structured manner on the secured server without any explicitly identifying information (name, ID number, email). Each participant will be assigned a coded participant number that will be used to identify the subject in the database. The code with the identified information will be stored in an encrypted form on a separate secured server that only the research manager will have access to. Access to all servers is restricted with username and password.

All (non-digital) questionnaires and signed informed consent documents will be stored in a secured cabinet in Tel Aviv University, to which only the research manager and the principal investigators will have access. No data collected as part of the study will be added to individuals' medical charts.

Data processing

We will perform several preprocessing steps. Concerning the daily questionnaires, in cases where participants will fill in the daily questionnaire more than once on a given day, only the last

entry for that day will be considered, as it is reasoned that the last one likely best represented the entire day. Self-reported symptoms that are entered as the free text will be manually categorized. With regard to the smartwatch physiological indicators, data will be aggregated per hour (by taking the mean value).

Data Analysis

We will define a "baseline period" as the 7 days prior to vaccination. We will consider a participant's "baseline" to be the last questionnaire they filled out during the baseline period for discrete measures or the entire baseline period for continuous measures. If a symptom was reported after vaccine administration and was not reported during the baseline period, we will assume this was a vaccine side effect. We will compare the baseline period to the "post-vaccination period," which will define as 7 and 14 days after vaccination, inclusive of vaccination day, for discrete and continuous metrics, respectively. To conduct the comparison will use standard statistical tools. Additionally, we will classify the participants into three major groups based on reported symptoms severity, and measure subjective and physiological responses in each group.

Potential Risks & Risk management

No specific risks arising from the smartwatches are expected, as the device is already commercialized with no known adverse reactions. The main risk in this study is the leakage of private data which we intend to manage as we describe in the following section.

Privacy/Confidentiality

Results from this study will be handled at an aggregated level. Individual data records will remain confidential and will not be published or shared with any third party. Signed and dated informed consent forms, as well as data recording sheets (e.g., case report forms) will be stored in locked cabinets during the study and following its completion. A file containing the personal details of the participants will be coded to help preserve confidentiality and will be separated from all other data collected throughout the study. This file will be kept by the principal investigator. Data will be stored on computers in password-protected files.

The data obtained from the smartwatch used in this study will be linked to a coded participant number. The smartwatch does not include a GPS. The data collected by the PerMed application will arrive directly to PerMed back-end servers and will be stored securely.

Supplementary References

1 Gepner, Y. *et al.* Utilizing wearable sensors for continuous and highly-sensitive monitoring of reactions to the BNT162b2 mRNA COVID-19 vaccine. *Commun. Med.* **2**, 27 (2022).

2 Mofaz, M. *et al.* Self-reported and physiological reactions to the third BNT162b2 mRNA COVID-19 (booster) vaccine dose. *Emerg. Infect. Dis.* **28**, 1375-1383 (2022).

3 Quer, G. *et al.* Wearable sensor data and self-reported symptoms for COVID-19 detection. *Nat. Med.* **27**, 73-77 (2021).

4 Laborde, S., Mosley, E. & Thayer, J. F. Heart rate variability and cardiac vagal tone in psychophysiological research - recommendations for experiment planning, data analysis, and data reporting. *Front. Psychol.* **8**, 213 (2017).

5 Porges, S. W. Cardiac vagal tone: a physiological index of stress. *Neurosci. Biobehav. Rev.* **19**, 225-233 (1995).

6 Oved, S. *et al.* Differential effects of COVID-19 lockdowns on well-being: interaction between age, gender and chronotype. *J. R. Soc. Interface.* **18**, 20210078 (2021).

7 Kim, H. G., Cheon, E. J., Bai, D. S., Lee, Y. H. & Koo, B. H. Stress and heart rate variability: a meta-analysis and review of the literature. *Psychiatry. Investig.* **15**, 235-245 (2018).

8 Pereira, T., Almeida, P. R., Cunha, J. P. S. & Aguiar, A. Heart rate variability metrics for fine-grained stress level assessment. *Comput. Methods. Programs. Biomed.* **148**, 71-80 (2017).

9 Bent, B., Goldstein, B. A., Kibbe, W. A. & Dunn, J. P. Investigating sources of inaccuracy in wearable optical heart rate sensors. *NPJ Digit. Med.* **3**, 18 (2020).

10 Reddy, R. K. *et al.* Accuracy of wrist-worn activity monitors during common daily physical activities and types of structured exercise: evaluation study. *JMIR Mhealth. Uhealth.* **6**, e10338 (2018).

Supplementary Figures

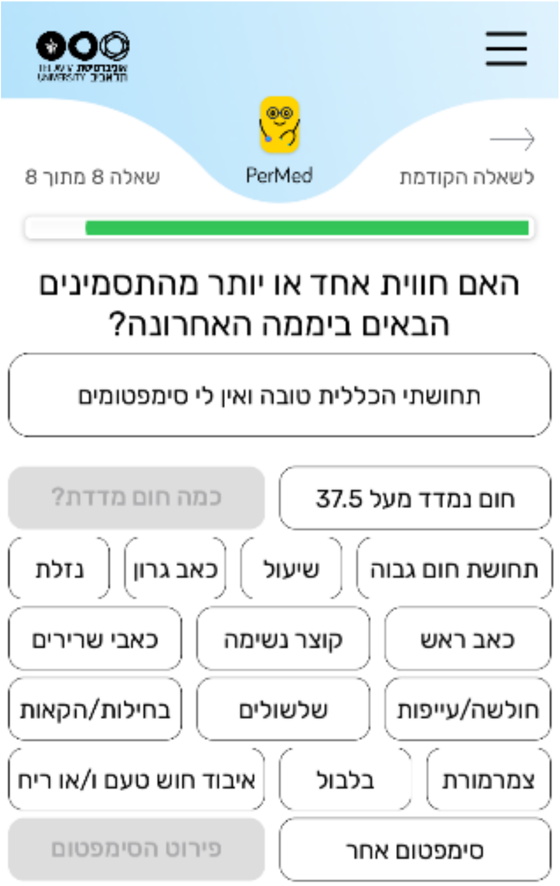

Supplementary Figure 1. Screenshot from mobile questionnaire showing the selection of symptoms. Translation: Have you experienced one or more of the following symptoms in the last 24 hours? My general feeling is good, and I have no symptoms, Heat measured above 37.5, Cough, Sore throat, Runny nose, Headache, Shortness of breath, Muscle aches, Weakness / fatigue, Diarrhea, Nausea / vomiting, Chills, Confusion, Loss of sense of taste / smell, Another symptom (fill in)

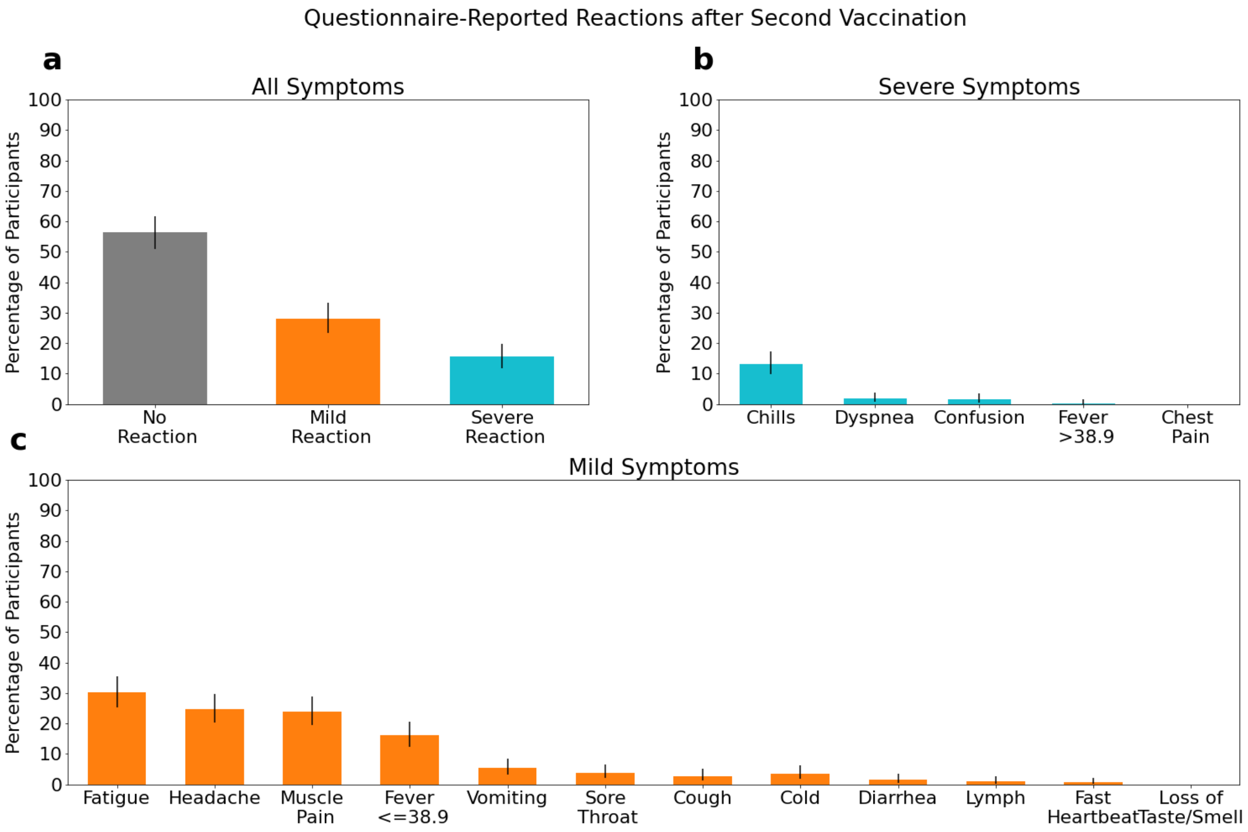

Supplementary Figure 2. Summary of symptoms as reported in the questionnaires after the second vaccination: Percentage of all participants classified into each severity tier based on their most severe reported symptom in the 72 hours following vaccination, and percentage of all participants reporting each of the severe and symptoms. Error bars represent 95% confidence intervals.

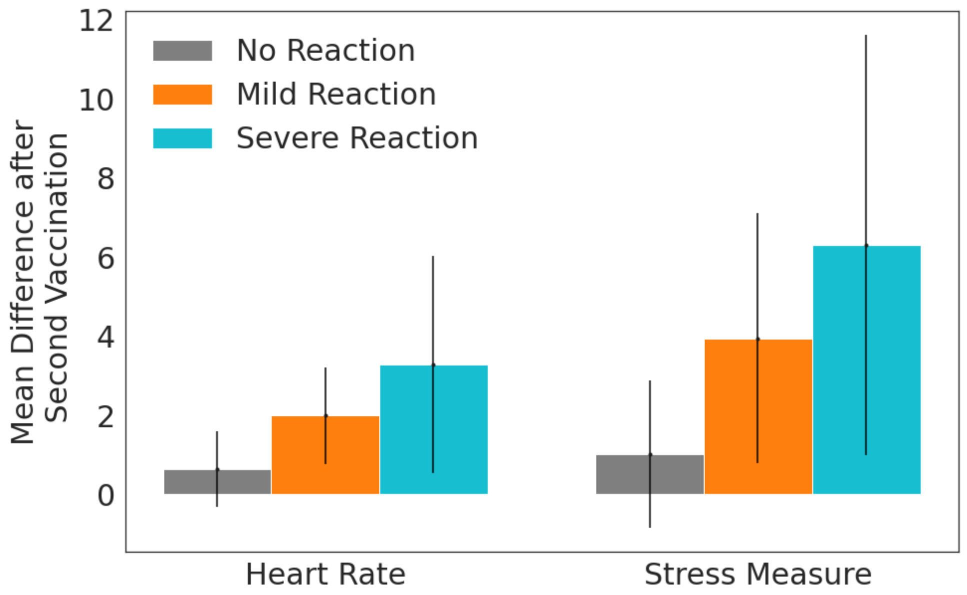

Supplementary Figure 3. Mean difference in heart rate (in beats per minute) and stress measure (in points) between the post-vaccination and baseline periods in Garmin smartwatch data in the 72 hours after the second vaccination, stratified by self-reported reaction severity. Error bars represent 95% confidence intervals.

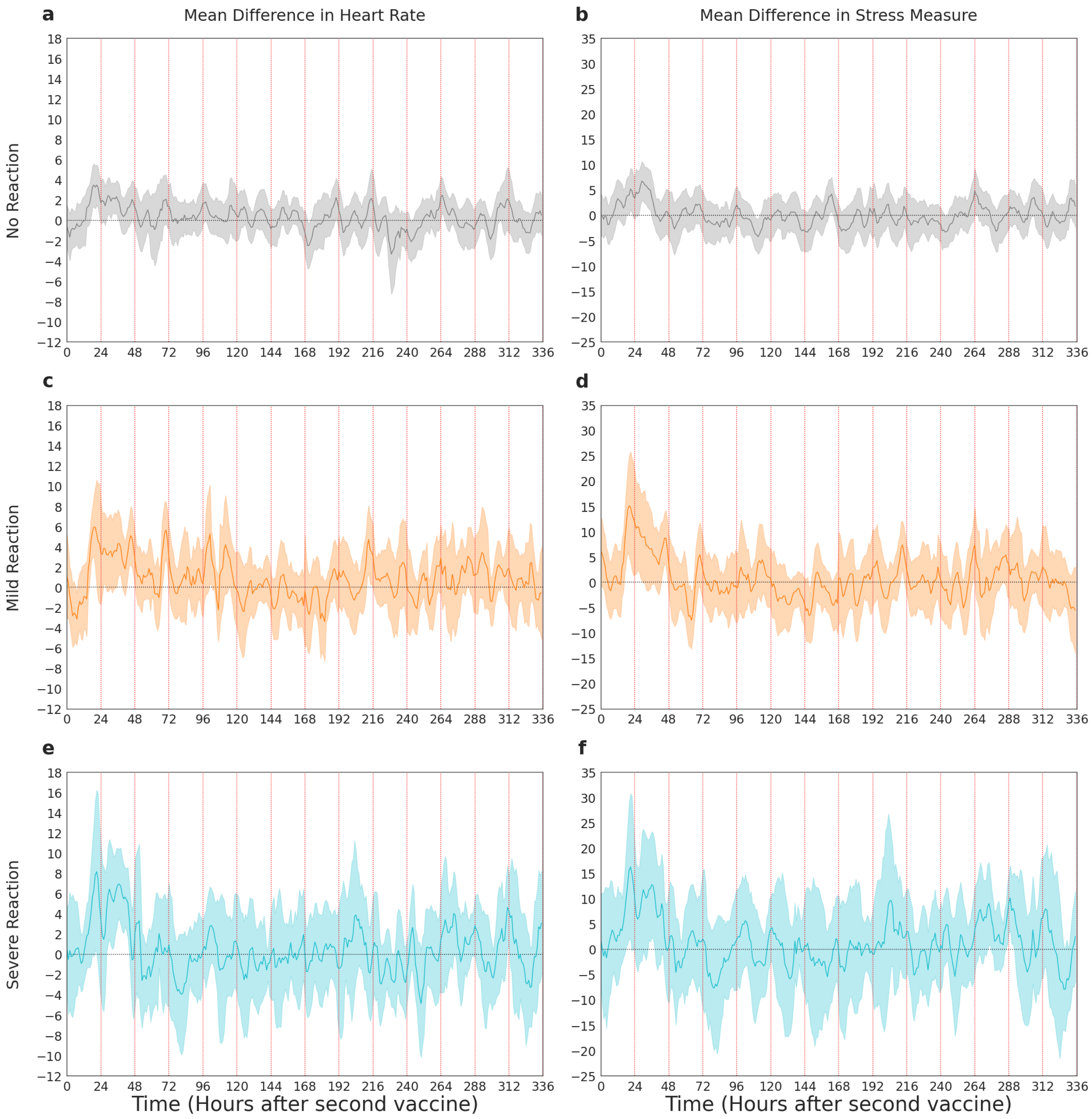

Supplementary Figure 4. Mean difference in heart rate (in beats per minute) and stress measure (in points) between the post-vaccination and baseline periods in Garmin smartwatch data after the second vaccination, by hour, for individuals who reported no reaction, mild reaction, and severe reaction in the self-reported questionnaires. Error bars represent 95% confidence intervals.

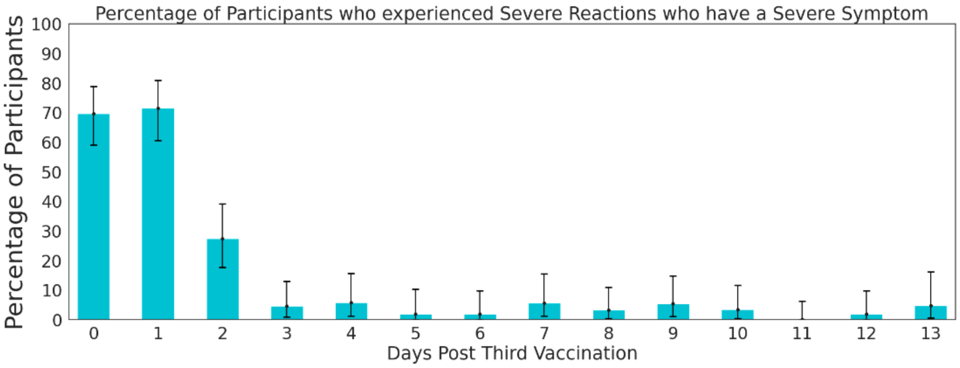

Supplementary Figure 5. Percentage of participants who experienced a severe reaction after the third vaccination who also reported at least one severe symptom in the days post-vaccination. Error bars represent 95% confidence intervals.

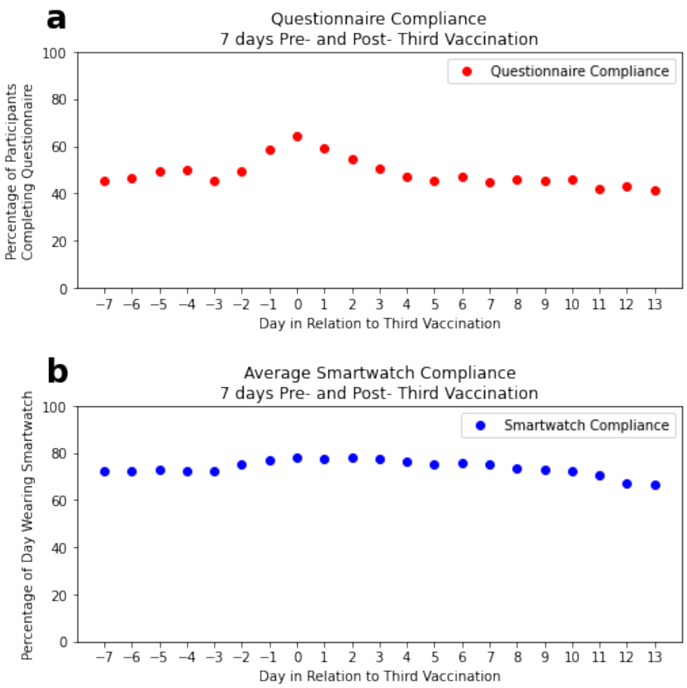

Supplementary Figure 6. Compliance with study protocol for 7 days prior to and 13 days after the third vaccination (where day 0 reflects the day of vaccination), reflecting the dates used in the panel regression. For questionnaire compliance (top), we assessed the percentage of participants completing the questionnaire each day out of the n=1,179 sample size reported in **Table 1**. Since participants were only required to fill out the questionnaire once every 3 days, the percentage of participants completing the questionnaire at least once in every 3 days from 7 days prior to and post vaccination was 89%. For smartwatch compliance (bottom), we assessed the percentage of each day each participant was wearing their smartwatch, only excluding participants who did not record any smartwatch measurements over this time period. All participants, even participants who did not wear their smartwatch on a given day, are included in this average.

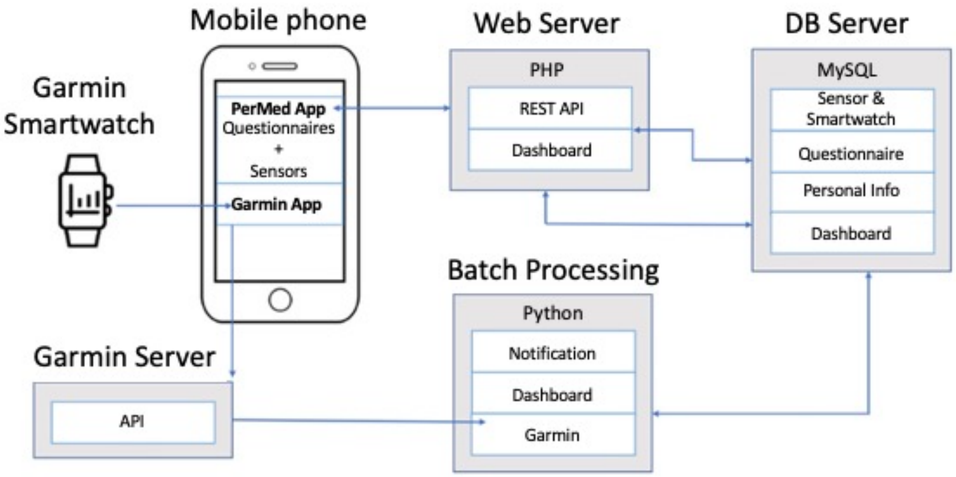

Supplementary Figure 7. The high-level architecture of the PerMed's data collection platform.

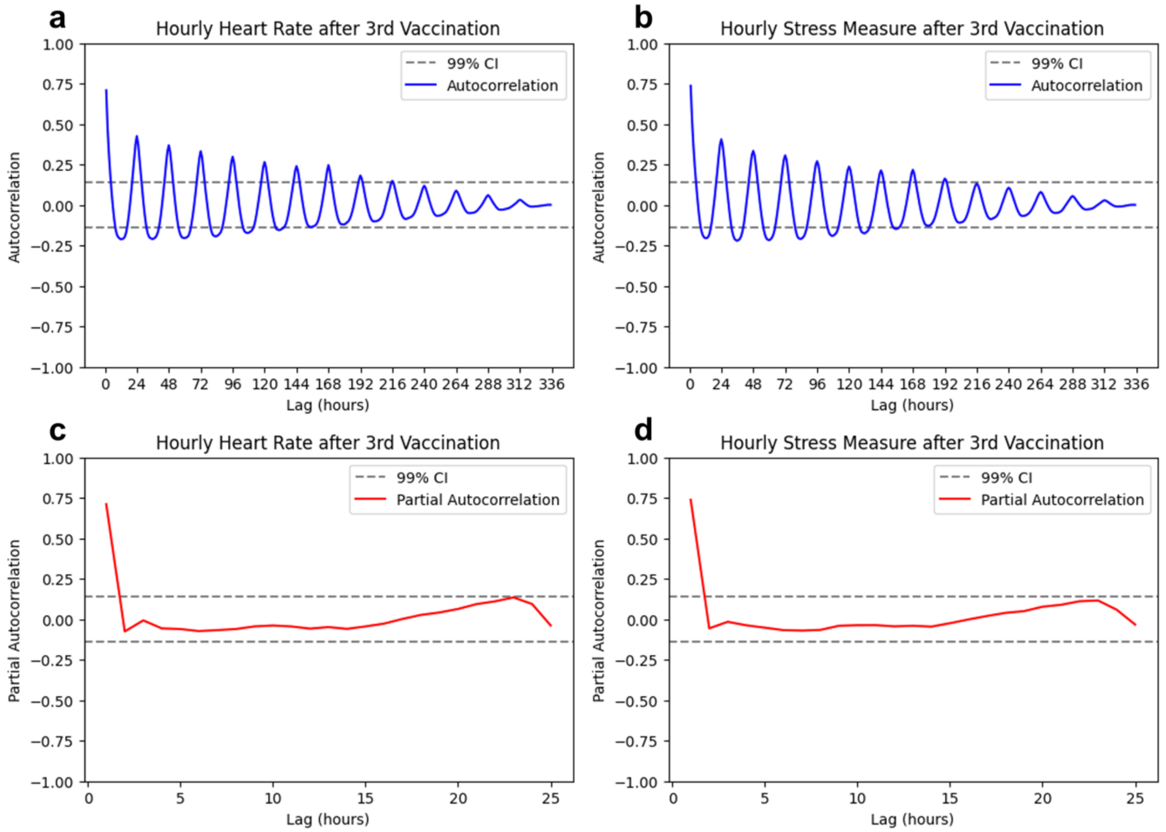

Supplementary Figure 8. Average autocorrelation (top) and partial autocorrelation (bottom) between hourly average measurements of heart rate (left) and stress measure (right) after the third vaccination. There was significant autocorrelation between each measurement and the measurement directly preceding. The correlation with the 24 hour lag was largely explained by lower-order lags.

Supplementary Table 1. Results of the mixed effects panel regression for all participants and asymptomatic participants only after the second vaccination. P-values for variable coefficients are from two-sided t-tests, and p-values from F-statistics are from F-tests.

Dependent Variable	Heart Rate (HR)					
	All Participants (n=163)			Asymptomatic Participants Only (n=81)		
Fixed and Random Effects	Coefficient	Std Err	p-value	Coefficient	Std Err	p-value
Between 0 and 72 hours after vaccination	0.7331	0.1045	<0.001	0.4007	0.1431	0.0051
HR or Stress in Previous Hour	0.4627	0.0031	<0.001	0.4771	0.0044	<0.001
Age	-0.0238	0.006	<0.001	0.0779	0.01	<0.001
No Underlying Medical Condition	-0.8243	0.136	<0.001	-0.5433	0.188	0.004
Male Gender	-3.7423	0.123	<0.001	-1.5258	0.174	<0.001
	F-statistic = 11040 (p-value<0.0001)			F-statistic = 6015 (p-value<0.0001)		
	R^2 Between = 0.6413			R^2 Between = 0.6726		
	R^2 Within =0.3128			R^2 Within =0.3231		
	R^2 Overall =0.4172			R^2 Overall =0.4440		
	% of Variance due to Random Effects: 6.14%			% of Variance due to Random Effects: 3.43%		
**Dependent Variable**	**Stress Measure[1]**					
	**All Participants (n=151)**			**Asymptomatic Participants Only (n=78)**		
**Fixed and Random Effects**	**Coefficient**	**Std Err**	**p-value**	**Coefficient**	**Std Err**	**p-value**
Between 0 and 72 hours after vaccination	0.6895	0.1478	<0.001	0.5793	0.2043	0.0046
HR or Stress in Previous Hour	0.7289	0.0032	<0.001	0.7165	0.0046	<0.001
Age	0.0105	0.009	0.266	0.184	0.014	<0.001
No Underlying Medical Condition	1.89	0.206	<0.001	2.5005	0.276	<0.001
Male Gender	0.0429	0.184	0.816	1.9956	0.258	<0.001
	F-statistic = 25790 (p-value<0.0001)			F-statistic = 12420 (p-value<0.0001)		
	R^2 Between = 0.8849			R^2 Between = 0.8521		
	R^2 Within =0.5202			R^2 Within =0.5191		
	R^2 Overall =0.5607			R^2 Overall =0.5562		
	% of Variance due to Random Effects: 0.29%			% of Variance due to Random Effects: 2.26%		

[1] The number of participants for the heart rate and stress measure panel regression differ slightly due to issues extracting the data from Garmin.

Supplementary Table 2. Percentage of participants complying with each step of the enrollment procedure as of September 15, 2021.

Enrollment procedure	Number of participants	Compliance percentage
Showed interest in participating the study and filled their contact information, and meet inclusion and exclusion criteria	5262	--
Showed interest in participating after a conversation with the study team	3637	69.1%
Enrolled in the study (signed informed consent form)	2476	47.0%
Active participants who remained in the study as of September 15 2021	2219	42.2%

Supplementary Table 3. Results of the mixed effects panel regression for asymptomatic participants after the third vaccination where the indicator variable has been changed to exclude the day of vaccination. P-values for variable coefficients are from two-sided t-tests, and p-values from F-statistics are from F-tests.

**Dependent Variable**	**Heart Rate (HR)**		
	**Asymptomatic Participants Only (n=523)**		
**Fixed and Random Effects**	**Coefficient**	**Std Err**	**p-value**
Between 24 and 72 hours after vaccination	0.383	0.0706	<0.001
HR or Stress in Previous Hour	0.4806	0.0018	<0.001
Age	-0.1638	0.003	<0.001
No Underlying Medical Condition	-1.6324	0.078	<0.001
Male Gender	-2.7722	0.075	<0.001
	F-statistic = 37580 (p-value<0.0001)		
	R^2 Between = 0.6640		
	R^2 Within = 0.3397		
	R^2 Overall = 0.4449		
	% of Variance due to Random Effects: 7.28%		
**Dependent Variable**	**Stress Measure**[2]		
	**Asymptomatic Participants Only (n=519)**		
	**Coefficient**	**Std Err**	**p-value**
Between 24 and 72 hours after vaccination	0.5793	0.1016	0.0013
HR or Stress in Previous Hour	0.737	0.0018	<0.001
Age	-0.1719	0.004	<0.001
No Underlying Medical Condition	1.7162	0.12	<0.001
Male Gender	1.1123	0.258	<0.001
	F-statistic = 87670 (p-value<0.0001)		
	R^2 Between = 0.8200		
	R^2 Within =0.5549		
	R^2 Overall =0.5826		
	% of Variance due to Random Effects: 4.11%		

[2] The number of participants for the heart rate and stress measure panel regression differ slightly due to issues extracting the data from Garmin.

Supplementary Table 4. Self-reported reaction severity after the second and third vaccinations for the 95 individuals who were in both cohorts and provided self-reported data.

		**Reaction to the Third Vaccination**		
		**Severe**	**Mild**	**None**
**Reaction to the Second Vaccination**	**Severe**	4	6	3
	**Mild**	0	8	8
	**None**	1	16	49
