# Supplementary material for: Higher sensitivity monitoring of reactions to COVID-19 vaccination using smartwatches
Source: NPJ Digit Med. 2022 Sep 9;5:140. doi: 10.1038/s41746-022-00683-w (PMC9461410; doi:10.1038/s41746-022-00683-w)
Supplement: Supplementary file 2 — Reporting Summary [file 41746_2022_683_MOESM2_ESM.pdf]

nature research

Corresponding author(s): Grace Guan
Last updated by author(s): August 16, 2022

Reporting Summary

Nature Research wishes to improve the reproducibility of the work that we publish. This form provides structure for consistency and transparency in reporting. For further information on Nature Research policies, see our Editorial Policies and the Editorial Policy Checklist.

Statistics

For all statistical analyses, confirm that the following items are present in the figure legend, table legend, main text, or Methods section.

n/a	Confirmed	
☐	☒	The exact sample size (n) for each experimental group/condition, given as a discrete number and unit of measurement
☐	☒	A statement on whether measurements were taken from distinct samples or whether the same sample was measured repeatedly
☐	☒	The statistical test(s) used AND whether they are one- or two-sided<br>*Only common tests should be described solely by name; describe more complex techniques in the Methods section.*
☐	☒	A description of all covariates tested
☐	☒	A description of any assumptions or corrections, such as tests of normality and adjustment for multiple comparisons
☐	☒	A full description of the statistical parameters including central tendency (e.g. means) or other basic estimates (e.g. regression coefficient) AND variation (e.g. standard deviation) or associated estimates of uncertainty (e.g. confidence intervals)
☐	☒	For null hypothesis testing, the test statistic (e.g. F, t, r) with confidence intervals, effect sizes, degrees of freedom and P value noted<br>*Give P values as exact values whenever suitable.*
☒	☐	For Bayesian analysis, information on the choice of priors and Markov chain Monte Carlo settings
☒	☐	For hierarchical and complex designs, identification of the appropriate level for tests and full reporting of outcomes
☒	☐	Estimates of effect sizes (e.g. Cohen's d, Pearson's r), indicating how they were calculated

Our web collection on statistics for biologists contains articles on many of the points above.

Software and code

Policy information about availability of computer code

Data collection	N/A
Data analysis	The code written for the study is publicly available at https://github.com/guanzgrace/vaccine-side-effects.

For manuscripts utilizing custom algorithms or software that are central to the research but not yet described in published literature, software must be made available to editors and reviewers. We strongly encourage code deposition in a community repository (e.g. GitHub). See the Nature Research guidelines for submitting code & software for further information.

Data

Policy information about availability of data

All manuscripts must include a data availability statement. This statement should provide the following information, where applicable:

- Accession codes, unique identifiers, or web links for publicly available datasets
- A list of figures that have associated raw data
- A description of any restrictions on data availability

The aggregated datasets analyzed for the study are publicly available at https://github.com/guanzgrace/vaccine-side-effects.

Field-specific reporting

Please select the one below that is the best fit for your research. If you are not sure, read the appropriate sections before making your selection.

☐ Life sciences ☒ Behavioural & social sciences ☐ Ecological, evolutionary & environmental sciences

For a reference copy of the document with all sections, see nature.com/documents/nr-reporting-summary-flat.pdf

Behavioural & social sciences study design

All studies must disclose on these points even when the disclosure is negative.

Study description	In this study we will analyze data that was already collected and will be collected as part of the PerMed study [1]. Participants in the PerMed study are recruited for a period of two years, during which they are equipped with a Garmin Vivosmart 4 smartwatches and are asked to wear them as much as they could. In addition, participants install two applications on their mobile phones: an application that passively collects data from the smartwatch and a dedicated mobile application which allows participants to fill a daily questionnaire and to report their vaccine date and specific hour.
Research sample	We study cohorts from a prospective observational trial of 355 and 1,179 individuals who had their second and third vaccinations, respectively, in Israel with the BNT162b2 mRNA (Pfizer BioNTech) COVID-19 vaccine between January 10, 2021, and September 15, 2021.
Sampling strategy	The sampling procedure was convenience sampling. Potential participants will be recruited through advertisements in social media, online banners, and word-of-mouth.
Data collection	Participants will be equipped with Garmin Vivosmart 4 smart fitness trackers. Among other features, the smartwatch provides all-day heart rate and heart rate variability and during-night blood oxygen saturation level tracking capabilities. All participants will complete the daily self-reported questionnaire in a dedicated application (the PerMed mobile application). The daily questionnaire we will use includes the following question:<br>Have you experienced one or more of the following symptoms in the last 24 hours?• My general feeling is good, and I have no symptoms• Heat measured above 37.5• Cough• Sore throat• Runny nose• Headache• Shortness of breath• Muscle aches• Weakness / fatigue• Diarrhea• Nausea / vomiting• Chills• Confusion• Loss of sense of taste / smell• Another symptom.
Timing	The participants whose data was analyzed in this study were vaccinated with the BNT162b2 mRNA (Pfizer BioNTech) COVID-19 vaccine between January 10, 2021, and September 15, 2021.
Data exclusions	No data were excluded.
Non-participation	No participants dropped out/declined participation.
Randomization	N/A

Reporting for specific materials, systems and methods

We require information from authors about some types of materials, experimental systems and methods used in many studies. Here, indicate whether each material, system or method listed is relevant to your study. If you are not sure if a list item applies to your research, read the appropriate section before selecting a response.

Materials & experimental systems

n/a	Involved in the study	
☒	☐	Antibodies
☒	☐	Eukaryotic cell lines
☒	☐	Palaeontology and archaeology
☒	☐	Animals and other organisms
☐	☒	Human research participants
☒	☐	Clinical data
☒	☐	Dual use research of concern

Methods

n/a	Involved in the study	
☒	☐	ChIP-seq
☒	☐	Flow cytometry
☒	☐	MRI-based neuroimaging

Human research participants

Policy information about studies involving human research participants

Population characteristics	See above.

Recruitment	To recruit participants and ensure they complete all the study's requirements, we will hire a professional survey company. Potential participants will be recruited through advertisements in social media, online banners, and word-of-mouth. The survey company is responsible for guaranteeing the participants meet the study's requirements, in particular, that the questionnaires are filled daily, ensuring the smartwatches are charged constantly and worn properly, and assisting participants resolve technical problems.
Ethics oversight	The study was approved by MHS' Helsinki institutional review board, protocol number 0122-20-MHS. All participants gave written informed consent to participate in the study and were advised both orally and in writing of the nature of the study. The study protocol is provided in the Supplement.

Note that full information on the approval of the study protocol must also be provided in the manuscript.
